# Impact of Chocolate Cadmium on Vulnerable Populations in Serbia

**DOI:** 10.3390/foods14010018

**Published:** 2024-12-25

**Authors:** Aleksandra Nešić, Milica Lučić, Jelena Vesković, Ljiljana Janković Mandić, Milan Momčilović, Andrijana Miletić, Antonije Onjia

**Affiliations:** 1 Vinča Institute of Nuclear Sciences, University of Belgrade, Mike Petrovića Alasa 12-14, 11351 Belgrade, Serbia; ljmandic@vin.bg.ac.rs; 2 Innovation Center of the Faculty of Technology and Metallurgy, 11120 Belgrade, Serbia; milica.lucic@tmf.bg.ac.rs; 3 Faculty for Technology and Metallurgy, University of Belgrade, Karnegijeva 4, 11120 Belgrade, Serbia; jveskovic@tmf.bg.ac.rs (J.V.); amiletic@tmf.bg.ac.rs (A.M.); 4 Faculty of Sciences and Mathematics, University of Niš, Višegradska 33, 18000 Niš, Serbia; milanmomcilovic@yahoo.com

**Keywords:** theobroma, cacao, milk, dark chocolate, health risk, non-carcinogenic, cancer, weekly intake, Monte Carlo, sensitivity

## Abstract

Chocolate is one of the most popular and widely consumed confectionery products. However, elevated cadmium (Cd) content in this commodity threatens food safety and human health. It is crucial to monitor the presence of Cd in chocolate and to evaluate its associated health risks. This study assessed the Cd levels in milk and dark chocolates from the Serbian market (*n* = 155). Cadmium concentrations varied between 0.010 and 0.29 mg/kg. The obtained values were used to evaluate the hazard quotient (HQ) and cancer risk (CR). The estimated weekly intakes (EWIs) were below the tolerable limits for all samples. However, in some samples, the EWI reached 60.9% and 63.5% of the tolerable limit for toddlers and other children, respectively. No health risk was found based on the HQ. On the other hand, based on CR values, all chocolate products can be classified as posing a moderate risk. The Monte Carlo simulation indicated that toddlers and other children were more exposed to non-carcinogenic risk, whereas vegetarians, adults, pregnant women, and other children were more exposed to cancer risk. Sensitivity analysis indicates that body weight, exposure frequency, and ingestion rate are the most influential factors for non-cancer and cancer health risks.

## 1. Introduction

Chocolate is produced from cocoa beans, which are seeds of the *Theobroma* cacao tree [1]. This confectionery is widely popular due to its pleasant flavor, but it is also renowned for its antioxidant and healthy properties [2,3]. Chocolate is a significant source of minerals and vitamins [4]. The tree originates from Central and South America, but it is also cultivated in Africa and Asia [1,5]. West Africa currently supplies 75% of all cocoa beans [6]. The most cultivated species are Forastero (*Theobroma cacao sphaerocarpum*), Criollo (*Theobroma cacao cacao*), and Trinitario (a hybrid of the two other groups) [7]. To be declared as chocolate, a product must contain at least 25% cacao solids (non-fat component) or 20% cacao butter (fat component) [8]. In bitter chocolates, the percentage of cocoa solids can range from 47% to 70%, 75%, or even more than 90% [9,10].

The concentration of heavy metals in food is a growing concern worldwide, as increased intake of these metals can lead to various health problems [11,12]. In the last few decades, due to human activities, the presence of Cd in the environment has increased significantly [13,14,15]. In general, Cd accumulates in nature through human activities such as mining, smelting, and industrial processes, and subsequently enters the food chain [11,12,13,14,15]. This has led to an increase in Cd levels in food, especially in animal entrails, cephalopods, mushrooms, cocoa, and rice [16]. The largest amount of Cd, about 90%, enters the human body through food, which is the most important source of contamination for the population, whereas the other 10% of Cd intake comes from the air and drinking water. One of the particular concerns is the presence of Cd in chocolates, where Cd can be absorbed by cocoa plants from contaminated soil, leading to its accumulation in cocoa beans and, ultimately, in chocolate products [17]. Several studies have shown that the Cd level in chocolate can vary depending on the region where the cocoa beans are grown, farming practices, and processing methods [18,19]. Exposure to Cd through contaminated food can lead to kidney and liver damage, bone diseases, and neurological effects [20,21]. The International Agency for Research on Cancer (IARC) has classified Cd as carcinogenic to humans (group 1) [22]. Therefore, it is extremely important to monitor the presence of Cd in both the starting raw materials and final products. The technological production of chocolate involves several operations that directly affect the quality of semi-products (cocoa mass and chocolate mass) as well as the quality of the finished product (chocolate) [1,6,23]. Previous studies have reported that Cd is distributed in non-fat fractions (cocoa solids), with higher Cd content in dark chocolate than in milk chocolate [5]. The primary factor influencing the Cd content of chocolate is the geographic origin of cocoa beans. Cocoa beans from Latin America generally have higher Cd levels than those from Asia and Africa [6]. Cadmium in cocoa beans can originate from naturally occurring Cd in the soil, fertilizers, and pesticides [5,19].

Health risk assessment of Cd in chocolate products is a public health concern because chocolates are a popular confectionary among people of all ages, especially among children, who are more susceptible to Cd poisoning [8]. The Commission Regulation (EU) 2023/915 prescribes the maximum residue level (MRL) of certain contaminants in food [24].

The aim of this work was to examine the Cd content in different chocolate samples using microwave digestion and inductively coupled plasma mass spectrometry (ICP-MS). The Cd levels were compared with those in chocolate from other studies and with the percentage of cacao content. Based on the obtained results, a health risk assessment was conducted by estimating the weekly intake of Cd and the non-carcinogenic and carcinogenic risks in different population groups. Additionally, a probabilistic approach was used to obtain more accurate health risk estimation.

## 2. Materials and Methods

### 2.1. Samples and Chemicals

Different chocolate products (*n* = 150) were purchased from a local market in Belgrade, Serbia, between March and June 2022. Chocolate products include 3 milk chocolates with cacao content ≤30%, 98 milk chocolates with cacao content ≥30 and <50%, and 49 dark chocolates with cacao content ≥50%. The ICP multi-element standard solution XXI for MS (MES-21–5) was purchased from AccuStandard (New Haven, CT, USA). Nitric acid (HNO_3_) for trace analysis (67–70%, *v*/*v*) and H_2_O_2_ (30%, *w*/*w*) were supplied from Fisher Chemical (Merelbeke, Belgium). Certified reference material (CRM) peach leaves (SRM 1547) were obtained from the National Institute of Standards and Technology (NIST, Gaithersburg, MD, USA).

### 2.2. Preparation of Samples

The homogenized chocolate sample (0.500 g) was placed into a PTFE tube, followed by the addition of 4 mL of 67–70% HNO_3_ and 1 mL of 30% H_2_O_2_. The mixture was digested using a microwave oven MARS 5 (CEM, Matthews, NC, USA) with the following regimen: 25 min to 120 psi, and held for 10 min. The digestate was then filtered and diluted with deionized water to 50 mL in a volumetric flask. All samples, blanks, and standards were analyzed in triplicate.

### 2.3. Instrumentation

Cadmium was quantified in chocolate products using inductively coupled plasma quadrupole mass spectrometry ICP-MS (Thermo Scientific iCAP Q, Winsford, UK), interfaced with a Cetac ASX-520 autosampler (Thermo Scientific, Omaha, NE, USA). The ICP-MS system was managed using Qtegra instrument control software 2.7. (Thermo Scientific, Winsford, UK), and the operating parameters of the instrument were: plasma power (RF), 1550 W; detector mode, pulse; nebulizer gas flow, 0.98 L/min; auxiliary gas flow, 0.84 L/min; cool gas flow, 14 L/min; collision gas flow, 4.6 mL/min. Other parameters were injector diameter, 2.5 mm ID; sample uptake/wash time, 45 s each; KED bias potential, 3 V; dwell time, 50 ns; sweeps, 15; the number of points per peak, 3; acquisition time, 3 × 50 s. The nebulizer gas was ultrapure argon (Ar) (purity ≥ 99.999%), while the collision gas was ultrapure helium (He) (purity ≥ 99.999%).

The calibration curve was constructed using eight calibration concentrations: 10, 20, 50, 100, 200, 500, 1000, and 5000 ng/L. The linear correlation coefficient (R^2^) was greater than 0.998, indicating good linearity of the calibration curve. The accuracy of the analytical procedures was confirmed by analyzing the SRM peach leaves (NIST SRM 1547) in triplicate for each digestion batch. Recovery ranged from 85% to 111%, and the relative standard deviation (RSD) was <11%. The limit of detection (LOD) and limit of quantification (LOQ) were defined as 3 and 10 times the standard deviation of the six procedural blanks divided by the slope of the analytical curve. The limit of quantification (LOQ) was 0.001 mg/kg.

### 2.4. Health Risk Assessment

#### 2.4.1. Estimated Weekly Cd Intake

The estimated weekly intake (EWI) and the percentage of tolerable weekly intake were calculated using the following equation:

(1) $$EWI={IngR}_{w}*C$$

(2) $$\%PTWI=\frac{EWI*100}{PTWI}$$

where IngR_w_ is the weekly ingestion rate of chocolate in g/kg bw per day, C is the concentration of Cd in µg/kg, and PTWI is the provisional tolerable weekly intake, as set by the European Food Safety Authority (EFSA), of 2.5 μg/kg body weight [25]. Data for ingestion rates (IngRs) were obtained from the EFSA Comprehensive Food Consumption Database, Exposure Hierarchy level 5 [26]. Since this database provides information on IngR in g/day and g/kg bw per day, we calculated the weekly ingestion. The IngRw values were obtained by multiplying the daily ingestion rate in g/kg bw per day by seven and then converting grams to kilograms. The calculation was performed for seven population groups (toddlers, other children, adolescents, adults, elderly, pregnant women, and vegetarians) based on the IngR in Serbia.

#### 2.4.2. The Non-Carcinogenic and Carcinogenic Risks

A parameter often used to assess the potential health risk associated with long-term exposure to a food (non-carcinogenic health risk) is the hazard quotient (HQ), proposed by the Environmental Protection Agency (USEPA), and can be calculated according to the following formula:

(3) $$HQ=\frac{EF\times ED\times C\times {IngR}_{d}\times CF}{{AT}_{nc}\times BW\times RfD}$$

where EF is the exposure frequency, set at 350 day(s)·year^−1^ and ED is the lifetime exposure duration, considered to be 2 years for toddlers (1–3), 5 years for other children (4–9 years), 7 years for adolescents (10–17 years), 30 years for adults, pregnant women, and vegetarians [27], and 15 years for the elderly [27,28,29,30]. C is the concentration of Cd in the chocolate sample (mg/kg), IngR_d_ is the daily ingestion rate (g/day), CF is the conversion factor (1 × 10^−3^ kg·mg^−1^), RfD is the oral reference dose of the element (mg·kg^−1^ ·day^−1^) [31], BW is the average body weight (kg), and AT_nc_ is the average time of exposure (365 days per year multiplied by exposure years, day(s)). Data for ingestion rates (IngR) were obtained from the EFSA Comprehensive Food Consumption Database, Exposure Hierarchy level 5 [26]. Since this database provides IngR in g/day and g/kg bw per day, in our calculation, we use IngR in g/kg bw per day, omitting BW from the equation. HQ values less than or equal to 1 indicate no adverse effects, while values greater than 1 are considered critical for health.

The cancer risk of Cd was calculated using Equation (4):

(4) $$CR=\frac{EF\times ED\times C\times {IngR}_{d}\times CF\times CSF}{{AT}_{c}\times BW}$$

EF, ED, C, IngR_d_, CF, and BW are the same as above, while AT_c_ is the average time for cancer risk and was 25,550 days for all population groups. The cancer slope factor (CSF) was 6.1 kg·day·mg^−1^ [32].

#### 2.4.3. Monte Carlo Simulations

The Monte Carlo simulation (MCS) was used to calculate the probabilistic health risks of Cd in chocolate. The simulation was performed with 10,000 repetitions in Oracle Crystal Ball^®^ (version 11.1.3.0.0, Oracle Inc., Redwood Shores, CA, USA). The probability distributions for the input parameters were as follows: C, lognormal; IngR, triangular; EF, triangular; ED, point; BW, lognormal; AT, point; CF, point; RfD, point; and CSF, point [32].

## 3. Results

### 3.1. Cadmium Concentration in Chocolates

**Figure 1:**
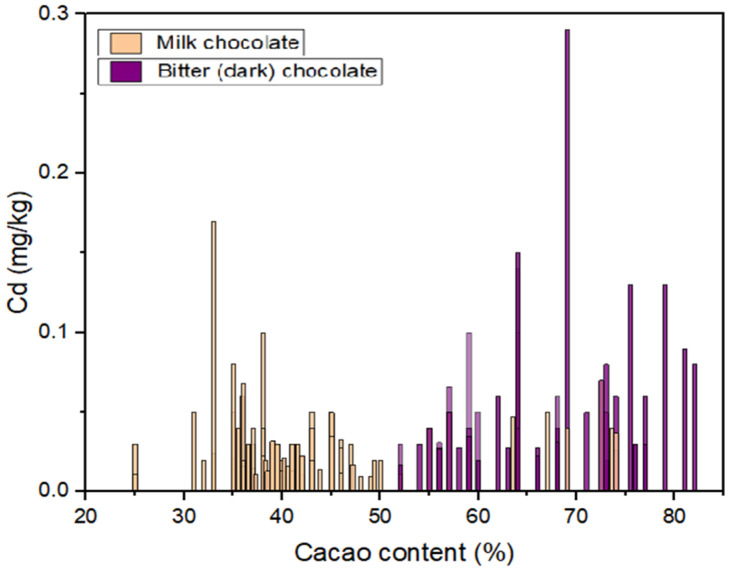
Cadmium content in chocolate samples vs. cacao content %.

The Cd concentrations detected in different chocolate samples are presented in Table 1. The relationship between cacao content and Cd content is illustrated in Figure 1. The chocolate samples are divided into three groups based on the percentage of cacao content and the maximum allowable level of Cd in these categories according to Commission Regulation (EU) 2023/915 [24]. The maximum allowable Cd content was not exceeded in the analyzed chocolate samples. The lowest and highest Cd levels in all chocolate products were 0.01mg/kg and 0.29 mg/kg, respectively. The mean concentrations were 0.024, 0.028, and 0.028 mg/kg in milk chocolates with cacao content <30, milk chocolates with cacao content ≥30 and <50, and bitter (dark) chocolates with cacao content ≥50%, respectively. It is important to note that Cd levels can vary significantly, even within the same brand or type of chocolate. Milk and dark chocolate are often made from the same cocoa beans, but the processing methods used to produce them can differ. Milk chocolate typically involves the addition of milk and sugar, which can dilute the Cd concentration [6,19]. However, the specific processing methods, cacao origin, and additives used can vary among manufacturers, leading to differences in Cd levels between milk and dark chocolate products. For example, Kruszewski et al. demonstrated that chocolate produced from cacao originating from Ecuador had a higher Cd content than chocolate produced from cacao originating from the Dominican Republic [19].

Figure 1 shows that dark chocolates generally had higher Cd content than milk chocolates, which is expected. However, there were a few milk chocolate samples with Cd concentrations similar to those found in dark chocolates. Although these Cd levels did not exceed the maximum allowable limit, the deviation from the other milk chocolate samples suggests either different sources of raw cacao or Cd from ingredients other than cocoa. In general, the geographic origin and percentage of cacao content are the two most influential factors on cadmium (Cd) content. Higher Cd levels in dark chocolates are associated with higher percentages of cocoa solids [6]. Previous studies have observed that cacao and chocolate products from Africa have lower Cd levels than those from Latin America [1,6].

Table 2 summarizes the Cd concentrations in chocolates from various markets based on the available literature. Besides the data about the market country, some studies also provide information on the origin of cacao (mainly the continent or region). Those data are given in brackets in the same column after the market country. The Cd levels detected in the milk chocolates were similar to those detected in the USA and Turkey in 2024 [4,17], India in 2016 [33], and Brazil in 2014 [8]. The Cd content in milk chocolates was significantly lower than that in some milk chocolates from Japan in 2018 [34] and India in 2005 [35], where the highest detected Cd concentrations were 0.310 and 0.852 mg/kg, respectively.

The Cd content of dark chocolates available on the Serbian market was found to be similar to chocolates from Serbia [36], the Dominican Republic [19], and Italy [37] in 2018. Similar results were also reported for dark chocolate in India [33] and Central America [2] in 2016, in Turkey in 2024 [4], Brazil in 2014 [8], Africa in 2016 [2], Malaysia in 2020 [38], and Lebanon in 2023 [5]. In contrast, dark chocolates from India in 2005 [35], Japan in 2018 [34], and the USA markets in 2024 [17] exhibited significantly higher Cd levels comparable to those observed in Serbian chocolates, with the highest detected Cd concentrations in dark chocolates being 2.73, 2.30, and 0.843 mg/kg, respectively.

**Table 2:** Comparison of Cd content in chocolate products from different markets.

Type of Chocolate	Number of Samples (*n*)	Country	Cd, mg/kg	Reference
Milk chocolate (cacao content <50%)	101	Serbia	0.013–0.17	This study
	50	United States	0.0003–0.161	[17]
	7	Africa †	0.0423 ± 0.0249	[23]
	12	Latin America †	0.0970 ± 0.0964	[23]
	21	India	0.140 ± 0.080	[33]
	1	Bosnia and Herzegovina	0.25	[39]
	35	Japan	0.012–0.31	[34]
	5	Croatia	0.00115–0.03394	[40]
	70	Italy	N.D.–0.050	[37]
	6	Turkey	0.141	[4]
	22	India	0.010–0.852	[35]
	9	Brazil	0.0078–0.0144	[8]
Black chocolate (cacao content > 50%)	49	Serbia	0.014–0.29	This study
	101	United States	0.0077–0.843	[17]
	8	Africa †	0.1054 ± 0.1073	[23]
	26	Latin America †	0.4247 ± 0.2924	[23]
	22	India	0.190 ± 0.270	[33]
	1	Ecuador *	0.429 ± 0.026	[19]
	1	Dominican Republic *	0.107 ± 0.012	[19]
	1	Bosnia and Herzegovina	0.50	[39]
	10	Serbia	0.025–0.242	[36]
	140	Japan	0.025–2.3	[34]
	7	Croatia	0.01895–0.1053	[40]
	35	Italy	0.019–0.207	[37]
	6	Turkey	0.209	[4]
	23	India	0.010–2.730	[35]
	11	Brazil	0.0221–0.1076	[8]
	1	West Africa **	0.144	[2]
	2	East Africa **	0.201 ± 0.031	[2]
	8	South America **	0.615 ± 0.398	[2]
	2	Central America **	0.142 ± 0.051	[2]
	35	Malaysia	0.01–0.2	[38]
	69	Lebanon ♦	0.023–0.352	[5]
	30	Lebanon ●	0.048–0.383	[5]
Not specified type of chocolate	33	Africa	0.20 ± 0.11	[1]
	14	Asia	0.12 ± 0.07	[1]
	22	Central America	0.33 ± 0.30	[1]
	69	South America	0.58 ± 0.52	[1]
	9	Saudi Arabia	0.0006–0.060	[41]
	-	Nigeria	<0.05–0.2	[42]

† US market; * Poland; ** Purchased in Italy, ♦ Production country Lebanon, ● Chocolates imported to Lebanon. N.D. – not detected.

In our study, the Cd content indicates that the cacao in these goods most likely comes from Africa and Asia, even if our analysis includes chocolates made and imported in Serbia, where the origin of the cacao is unknown. Previous studies found that chocolates from South America contained significantly higher Cd concentrations than those from Africa and Asia [1]. Generally, cocoa beans and chocolates originating from Latin America tend to have higher Cd content than those from Africa and Asia [6]. However, there are exceptions in some studies, such as certain samples with cacao from Latin America (U.S. market) [23] and Central America (Italy market) [2], where elevated cadmium content was not observed. Chocolates are usually made by combining cacao beans of different origins, leading to significant variations in Cd content between different chocolates [1,4]. However, single-origin products, including chocolates of single origin, are gaining popularity and are often marketed as luxury products [1].

The geographical origin of cocoa beans and the specific processing methods used can significantly influence the heavy metal content of chocolate. Factors such as soil contamination, fertilizer use, and roasting conditions can contribute to the accumulation of heavy metals in cocoa beans. To ensure the safety of chocolate products, it is essential to implement effective monitoring and surveillance programs to assess the prevalence of heavy metals. Additionally, research should focus on identifying and mitigating factors that contribute to the contamination of cocoa beans.

### 3.2. Health Risk Assessment

The safety of chocolate consumption was estimated based on weekly chocolate consumption by consumers in Serbia and the concentration of Cd in the analyzed samples. The weekly milk chocolate consumption for toddlers, children, adolescents, adults, pregnant women, and the elderly in Serbia was 0.00703, 0.00589, 0.00350, 0.00259, 0.00179, 0.00186, and 0.00322 kg/kg bw per week, respectively. The weekly bitter chocolate consumption for toddlers, children, adolescents, adults, pregnant women, and the elderly was 0.00525, 0.00547, 0.00157, 0.00154, 0.00134, 0.00214, and 0.00211 kg/kg bw per week, respectively. The European Food Safety Agency (EFSA) established a provisional tolerable weekly intake (PTWI) of Cd of 2.5 μg/kg body weight. The estimated weekly intake (EWI) of Cd was compared with the PTWI values and expressed as a percentage of PTWI. The min, max, and mean EWIs and corresponding %PTWI values are presented in Supplementary Material Table S1. The %PTWI for all analyzed samples was lowest for the elderly (from 0.72% to 15.6%) and highest for toddlers (from 2.81% to 60.9%) and other children (from 2.35% to 63.5%). The highest EWI of Cd in all population groups was for bitter chocolate with 72% cacao and milk chocolate with whole hazelnuts. Based on the assessment of weekly cadmium intake, it can be concluded that most samples do not pose a risk because chocolate consumption does not exceed the PTWI values. However, it should be noted that chocolate is not the only source of Cd. Therefore, this food product should be consumed in limited quantities, especially in children, where it has been shown that the intake of certain dark chocolates exceeds 60% of the PTWI of Cd.

Table 3 presents the health risk analysis results for the seven population groups. The hazard quotient (HQ) describes the non-carcinogenic risk associated with Cd exposure. This parameter is also linked to the health risk of long-term exposure to the contaminant. If the Cd level in the chocolate exceeds an HQ of 1, the chocolate is not safe for consumption. On the other hand, values below 1 indicate that chocolate is safe for consumption. The mean HQ values were 2.69 × 10^−2^, 2.37 × 10^−2^, 1.20 × 10^−2^, 9.40 × 10^−3^, 7.91 × 10^−3^, 8.14 × 10^−3^ and 1.19 × 10^−2^ for toddlers, other children, adolescents, adults, elderly, pregnant women, and vegetarians, respectively. In our study, all maximum HQ values were below 1, indicating that chocolates from the Serbian market are safe for consumption in all examined population groups, considering the non-carcinogenic risk from Cd. Similar findings have been confirmed for dark chocolate [5], cacao powder [43], and white, milk, and dark chocolates [41] by other authors. For cancer risk (CR) analysis, there is no risk of cancer formation in humans when CR is at 1 × 10^−6^ or below. Risks are acceptable or tolerable in the range of 1 × 10^−6^ to 1 × 10^−4^. If the CR value surpasses 1 × 10^−4^, the risk is unacceptable [32]. In our study, the mean CR values were 5.81 × 10^−6^, 1.54 × 10^−5^, 8.07 × 10^−6^, 2.83 × 10^−5^, 1.29 × 10^−5^, 2.75 × 10^−5^, 3.64 × 10^−5^ for toddler, other children, adolescents, adults, elderly, pregnant women, and vegetarians, respectively. Those values greater than 1 × 10^−6^ indicate that chocolate products pose a moderate risk due to Cd content. This study revealed that all chocolate samples pose a moderate risk for toddlers, adolescents, and the elderly. For other children, adults, pregnant women, and vegetarians, 99.3%, 98.7%, 96.0%, and 96.7% of the analyzed samples, respectively, indicated a moderate risk. In the latter groups, 0.7%, 1.3%, 4%, and 3.3% of chocolates posed unacceptable risks.

**Figure 2:**
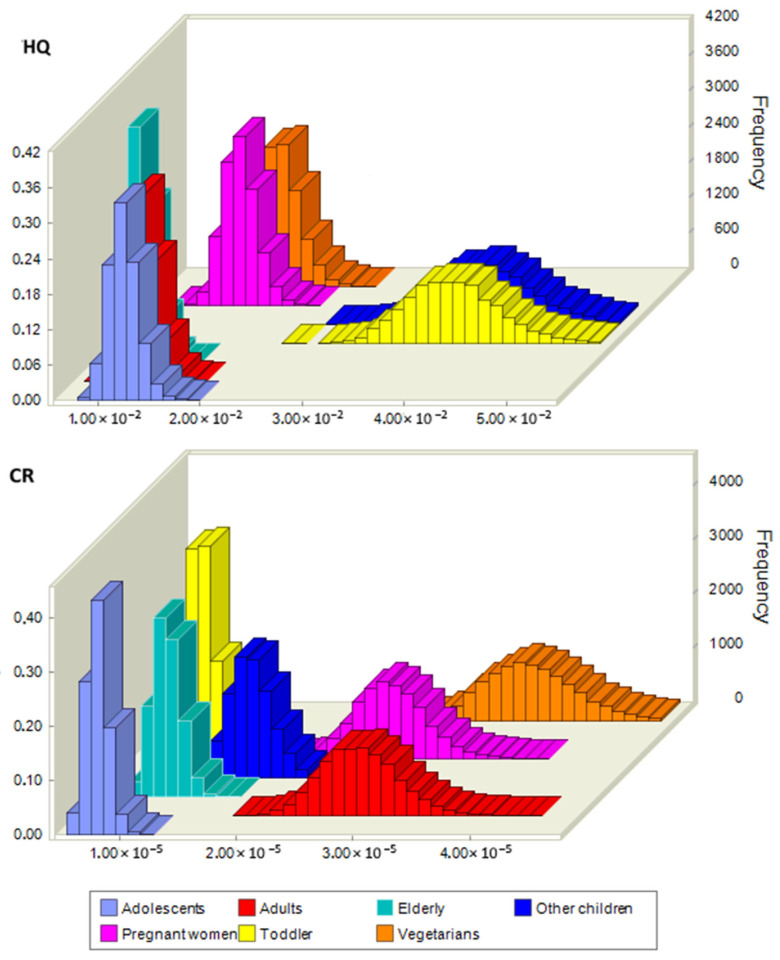
Monte Carlo simulation of HQ and CR for various consumers of chocolate due to Cd ingestion. The 90th percentiles were as follows: for adolescents HQ = 1.4 × 10^−2^ and CR =9.3 × 10^−6^; for adults HQ = 1.4 × 10^−2^ and CR =3.3 × 10^−5^; for elderly HQ = 1.2 × 10^−2^ and CR =1.2 × 10^−5^; for other children HQ = 4.7 × 10^−2^ and CR =1.9 × 10^−5^; for pregnant women HQ = 1.7 × 10^−2^ and CR =3.1 × 10^−5^; for toddlers HQ = 4.5 × 10^−2^ and CR =1.0 × 10^−5^ and for vegetarians HQ = 1.9 × 10^−2^ and CR =4.1 × 10^−5^.

A probabilistic health risk assessment of Cd for chocolate consumption was performed using a Monte Carlo simulation. Similar to the deterministic approach, we considered seven consumer groups: toddlers, other children, adolescents, adults, the elderly, pregnant women, and vegetarians. The scenario involved the ingestion rate of chocolate for consumers, as in the deterministic health risk assessment, i.e., we did not use ingestion rates for the average population. The probability distributions of health risks are presented in Figure 2. All HQ values were below the limit, indicating that Cd in the analyzed chocolates did not pose a non-carcinogenic risk to human health. Compared with other population groups, toddlers and other children were more exposed to non-carcinogenic risk. The 90th percentiles were 4.52 × 10^−2^ and 4.69 × 10^−2^ for toddlers and other children, respectively. For carcinogenic risk, the 90th percentiles were above 1.00 × 10^−6^ for all population groups, indicating a moderate risk. The highest risk groups for carcinogenic effects were vegetarians, adults, pregnant women, and other children.

**Figure 3:**
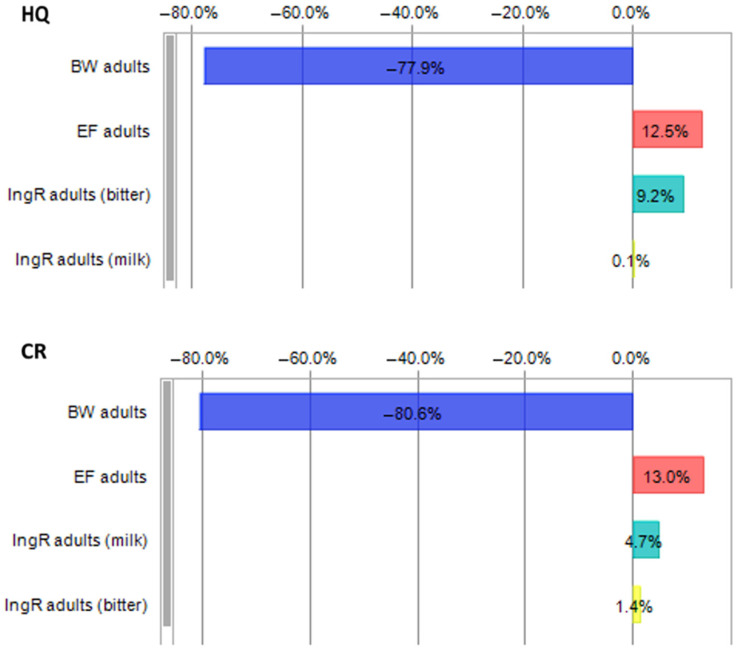
Sensitivity analysis of HQ and CR for adults due to ingestion of Cd via chocolate consumption.

The sensitivity analysis revealed that the two most influential factors responsible for health hazards were BW and EF. This was the case for all population groups, except for carcinogenic risk in toddlers, for which the second most influential factor was the ingestion rate of bitter chocolate. The sensitivity chart for non-carcinogenic and carcinogenic health risk assessment in adults is presented in Figure 3. The most influential parameters for other population groups are presented in Supplementary Material Table S2. For all population groups, the influence of factors on non-carcinogenic risk was as follows: BW > EF > IngR bitter > IngR milk. The impact of this factor on carcinogenic risk was also the same in pregnant women. In the case of carcinogenic risk, the factor influencing five population groups (other children, adolescents, adults, elderly, and vegetarians) was as follows: BW > EF > IngR milk > IngR bitter. As previously mentioned, the exceptions were the influence of factors on health risks for toddlers and pregnant women. For toddlers, the influence was as follows: BW > IngR bitter > EF > IngR milk, whereas for pregnant women, BW > EF > IngR bitter > IngR milk.

## 4. Conclusions

This study revealed variability in Cd content among the chocolate samples. Cadmium content largely depended on the percentage of cacao content, resulting in higher Cd levels in dark chocolates. The health risk assessment indicated that the weekly intake of certain chocolate samples could be risky for toddlers and other children, representing 60.9% and 63.5% of the provisional tolerable weekly intake (PTWI), respectively. The hazard quotient (HQ) indicated that chocolate consumption does not pose a non-carcinogenic risk, whereas the cancer risk (CR) indicated that chocolate consumption poses a moderate risk for all populations (toddlers, other children, adolescents, adults, elderly, pregnant women, vegetarians).

Monte Carlo simulations (MCSs) revealed that chocolate consumption poses a higher non-carcinogenic risk to toddlers and other children compared to other population groups. Regarding cancer risk, the MCS indicated that vegetarians, adults, pregnant women, and other children were more exposed to cancer risk than other groups. Continuous monitoring of Cd levels in chocolates is necessary because of the moderate cancer risk and increased risk for toddlers and children compared with other groups. The general conclusion is that chocolate consumption poses a greater risk for children, so it is important to monitor their intake of this treat.

## Figures and Tables

**Table 1:** Cadmium content in milk and dark chocolates, and MRL values for cadmium according to Commission Regulation (EU) 2023/915.

Type of Chocolate	Cacao Content, %	*n*	Cd (Min–Max) mg/kg	Mean Cd., mg/kg	Median, mg/kg	MRL, mg/kg
Milk chocolate	<30	3	0.013–0.030	0.024	0.031	0.1
Milk chocolate	≥30 and <50	98	0.010–0.17	0.028	0.023	0.3
Dark chocolate	≥50	49	0.014–0.29	0.057	0.042	0.8

**Table 3:** Hazard quotients (HQs) and cancer risks (CRs) for Cd from milk and dark chocolates (mean, min, and max values).

	Toddlers (1–3 Years)	Other Children (4–9 Years)	Adolescents (9–17 Years)	Adults (18–64 Years)	Elderly (>64 Years)	Pregnant Women	Vegetarians
HQ mean	2.69 × 10^−2^	2.37 × 10^−2^	1.20 × 10^−2^	9.40 × 10^−3^	7.91 × 10^−3^	8.14 × 10^−3^	1.19 × 10^−2^
HQ min	9.68 × 10^−3^	8.05 × 10^−3^	2.95 × 10^−3^	2.95 × 10^−3^	2.68 × 10^−3^	2.59 × 10^−3^	4.03 × 10^−3^
HQ max	1.65 × 10^−1^	1.37 × 10^−1^	8.15 × 10^−2^	6.03 × 10^−2^	4.56 × 10^−2^	4.40 × 10^−2^	7.50 × 10^−2^
CR mean	5.81 × 10^−6^	1.54 × 10^−5^	8.07 × 10^−6^	2.83 × 10^−5^	1.29 × 10^−5^	2.75 × 10^−5^	3.64 × 10^−5^
CR min	2.53 × 10^−6^	4.21 × 10^−6^	1.80 × 10^−6^	7.72 × 10^−6^	3.51 × 10^−6^	6.77 × 10^−6^	1.05 × 10^−5^
CR max	1.72 × 10^−5^	1.13 × 10^−4^	4.97 × 10^−5^	1.60 × 10^−4^	9.45 × 10^−5^	2.25 × 10^−4^	2.18 × 10^−4^

## Data Availability

The data that support the findings of this study are available from the corresponding author upon reasonable request.

## Supplementary Materials

The following supporting information can be downloaded at: https://www.mdpi.com/article/10.3390/foods14010018/s1, Table S1: Estimated weekly intake (EWI) of Cd through consumption of chocolate and its respective provisional tolerable weekly intake (PTWI) percentages for various population groups; Table S2: Influence of various parameters on HQ and CR according to sensitivity analysis for ingestion of Cd via chocolate consumption.
