# Supplementary material for: Impact of Chocolate Cadmium on Vulnerable Populations in Serbia
Source: Foods. 2024 Dec 25;14(1):18. doi: 10.3390/foods14010018 (PMC11719608; doi:10.3390/foods14010018)
Supplement: Supplementary file 1 [file foods-14-00018-s001.zip › foods-3353312-supplementary.pdf]

Supplementary material

Impact of Chocolate Cadmium on Vulnerable Populations in Serbia

Aleksandra Nešić [1*], Milica Lučić [2], Jelena Vesković [3], Ljiljana Janković Mandić [1], Milan Momčilović [4], Andrijana Miletić [3], Antonije Onjia [3,*]

1 Vinča Institute of Nuclear Sciences, University of Belgrade, Mike Petrovića Alasa 12-14, 11351 Belgrade, Serbia, anesic@vin.bg.ac.rs, ljmandic@vin.bg.ac.rs
2 Innovation Center of the Faculty of Technology and Metallurgy, 11120 Belgrade, Serbia; milica.lucic@tmf.bg.ac.rs
3 Faculty for Technology and Metallurgy, University of Belgrade, Karnegijeva 4, 11120 Belgrade, Serbia, onjia@tmf.bg.ac.rs; jveskovic@tmf.bg.ac.rs; amiletic@tmf.bg.ac.rs
4 Faculty of Sciences and Mathematics, University of Niš, Višegradska 33, 18000 Niš, Serbia, milanmomcilovic@yahoo.com
* Correspondence: anesic@vin.bg.ac.rs , onjia@tmf.bg.ac.rs

Table S1. Estimated weekly intake (EWI) of Cd through consumption of chocolate and its respective provisional tolerable weekly intake (PTWI) percentages for various population groups.

	Toddlers (1-3)	Other Children (4-9)	Adolescents (10-17)	Adults (18-64)	Eldery	Pregnant women	Vegaterians
EWI (min) μg/kg bw, per week	0.0704	0.0589	0.0220	0.0216	0.0180	0.0186	0.0296
EWI (max) μg/kg bw, per week	1.52	1.59	0.595	0.447	0.390	0.620	0.613
EWI (mean) μg/kg bw, per week	0.231	0.214	0.0960	0.0780	0.0591	0.0751	0.101
% PTWI (min)	2.81	2.35	0.88	0.86	0.72	0.74	1.18
% PTWI (max)	60.9	63.5	23.8	17.9	15.6	24.8	24.5
% PTWI (mean)	9.26	8.55	3.84	3.12	2.37	3.00	4.02

Table S2. Influence of various parameters on HQ and CR according to the sensitivity analysis for ingestion of Cd via chocolate consumption.

The most influential parameter	Sensitivity analysis (%)	The most influential parameter	Sensitivity analysis (%)
CR		HQ	
Toddler			
BW	-75.7	BW	-77.4
IngR bitter	12.7	EF	11.8
EF	11.5	IngR bitter	10.6
IngR milk	0.0	IngR milk	0.1
Other children			
BW	-81.0	BW	-77.7
EF	12.4	EF	11.9
IngR milk	3.9	IngR bitter	10.0
IngR bitter	2.4	IngR milk	0.1
Adolescents			
BW	-79.6	BW	-77.4
EF	13.0	EF	13.0
IngR milk	5.9	IngR bitter	9.1
IngR bitter	1.2	IngR milk	0.2
Adults			
BW	-80.6	BW	-77.9
EF	13.0	EF	12.5
IngR milk	4.7	IngR bitter	9.2
IngR bitter	1.4	IngR milk	0.1
Elderly			
BW	-79.2	BW	-76.0
EF	13.4	EF	12.9
IngR milk	4.5	IngR bitter	10.7
IngR bitter	2.5	IngR milk	0.1
Pregnant women			
BW	-80.0	BW	-76.3
EF	14.0	EF	13.4
IngR bitter	2.9	IngR bitter	10.1
IngR milk	2.8	IngR milk	0.0
Vegetarians			
BW	-80.8	BW	-76.7
EF	13.5	EF	12.9
IngR milk	3.1	IngR bitter	10.1
IngR bitter	2.3	IngR milk	0.0
